# The EG95 Antigen of *Echinococcus* spp. Contains Positively Selected Amino Acids, which May Influence Host Specificity and Vaccine Efficacy

**DOI:** 10.1371/journal.pone.0005362

**Published:** 2009-04-29

**Authors:** Karen Luisa Haag, Bruno Gottstein, Francisco Jose Ayala

**Affiliations:** 1 Departamento de Genética, Instituto de Biociências, Universidade Federal do Rio Grande do Sul, Porto Alegre, Brazil; 2 Institute of Parasitology, Faculty of Medicine and Veterinary Medicine, University of Berne, Berne, Switzerland; 3 Department of Ecology and Evolutionary Biology, University of California Irvine, Irvine, California, United States of America; Georgia Institute of Technology, United States of America

## Abstract

Echinococcosis is a worldwide zoonotic parasitic disease of humans and various herbivorous domestic animals (intermediate hosts) transmitted by the contact with wild and domestic carnivores (definitive hosts), mainly foxes and dogs. Recently, a vaccine was developed showing high levels of protection against one parasite haplotype (G1) of *Echinococcus granulosus*, and its potential efficacy against distinct parasite variants or species is still unclear. Interestingly, the EG95 vaccine antigen is a secreted glycosylphosphatydilinositol (GPI)-anchored protein containing a fibronectin type III domain, which is ubiquitous in modular proteins involved in cell adhesion. EG95 is highly expressed in oncospheres, the parasite life cycle stage which actively invades the intermediate hosts. After amplifying and sequencing the complete CDS of 57 *Echinococcus* isolates belonging to 7 distinct species, we uncovered a large amount of genetic variability, which may influence protein folding. Two positively selected sites are outside the vaccine epitopes, but are predicted to alter protein conformation. Moreover, phylogenetic analyses indicate that EG95 isoform evolution is convergent with regard to the number of beta-sheets and alpha-helices. We conclude that having a variety of EG95 isoforms is adaptive for *Echinococcus* parasites, in terms of their ability to invade different hosts, and we propose that a mixture of isoforms could possibly maximize vaccine efficacy.

## Introduction

Adult stages of the genus *Echinococcus* are small flatworms (2–7 mm-long), which live in the intestine of carnivores, usually wild and domestic dogs, wolves and foxes. The larvae (metacestodes) reproduce asexually, generating protoscoleces, and frequently develop in the liver and lungs of various herbivorous mammals. Intermediate herbivorous hosts become infected by the ingestion of eggs released with the feces of a parasitized carnivore. The parasite is transmitted to the definitive host via predator-prey interactions. Phylogenetic studies upon complete mitochondrial genomes inferred that *Echinococcus* species probably begun to diverge during the Miocene, following the diversification of large mammals [1]. The most ancestral species seems to be *E. oligarthrus*, a Neotropical parasite of felids and rodents. The other species develop in canids during the adult (sexual) stage, and several species of rodents (*E. multilocularis* and *E. vogeli*), lagomorphs (*E. multilocularis*), artiodactils (*E. granulosus* and *E. ortleppi*) and perissodactils (*E. equinus*) during the larval stage. Animal domestication by human societies strongly influenced *Echinococcus* spp. evolution. The parasite evolved into highly divergent strains and species, adapted to pig, cattle, sheep, camels, horses, and was subsequently spread throughout the world with the expansion of human populations and animal trade.

A critical aspect in the transmission cycle, and in the adaptation to different intermediate host species, is the ability of *Echinococcus* activated oncospheres, after hatching from the eggs, to penetrate the intestine cell wall and to reach the target organ of the herbivorous host via blood and lymph vessels [2]. Recently, a highly immunogenic protein was characterized, named EG95 for *E. granulosus*, and EM95 for *E. multilocularis*, which may be involved in host invasion, and is encoded by a multigene family [3], [4]. The antigen is a secreted protein with a GPI anchor and a fibronectin type III (Fn3) domain [5], upregulated during oncosphere activation [6], [7], and probably involved in cell adhesion [8]. Fn3 domains are usually involved in cell surface binding.

The interest on EG95 comes largely from its high potential as a vaccine to protect against *Echinococcus* infection. Related antigens from *Taenia* (45W, 18K and 16K), showing a similar molecular structure and expression pattern [9] provide similar levels of protection against cysticercosis (the disease caused by *Taenia solium*). Protection obtained with the recombinant antigen ranges from 83% (EM95) to 100% (EG95; reviewed in [10], [11]), and the elicited immune response effectively kills oncospheres *in vitro* [12]. The fact that EG95-related antigens are so immunogenic is puzzling, since the Fn3 module contains highly conserved features, and is one of the most widespread domains of mosaic proteins [13], being present both in the parasite and the host. Indeed, although the overall structure of the human leukocyte common antigen (CD45) contains conserved features, the exons encoding the Fn3-domain indicate an extraordinarily strong diversifying selection during the evolution of Old World monkeys, apes and humans [14].

Seven members of the *eg95* multigene family were isolated so far (*eg95-1* to *7*), showing a highly conserved structure, with three exons separated by two introns [3]. Exon 1 encodes a signal peptide, exons 2 and 3 encode the mature secreted protein, with most of the Fn3-domain being encoded by exon 2. A third intron occurs in the 3′ untranslated region, and one family member (*eg95-7*) is a pseudogene. Little is known about the diversity of genes encoding EG95 and EM95. Zhang et al [7] cloned cDNAs derived from pooled individuals, separated by life cycle stages, and found a very limited amount of variation, predominantly outside the protective epitopes. However, since the parasite materials were pooled, obtained from a single geographic area, and since there was no attempt to assess genetic variation among isolates derived from different strains or species, the data are not useful for predicting vaccine efficacy. Furthermore, it is known that the EG95 epitopes are conformational [15], [16], implying that a small number of amino acid replacements may affect protection dramatically.

Searching for adaptive amino acids would clarify two important aspects concerning the evolution of EG95 and EM95. First, the pattern and amount of adaptive evolution allows predictions about the potential vaccine efficacy over time and space. If *Echinococcus* strains or species from different geographic areas contain positively selected amino acids within the protective epitopes, one would expect a lower efficacy, because good vaccine epitopes should be rich in negatively selected sites [17]. Second, it would also bring some clues about how *Echinococcus* parasites evolved the ability to infect so many different intermediate host species during the last 40 million years. Variations in the Fn3-domain folding, allowing oncospheres to bind variant host cell receptors, might have been adaptive for the specific invasion of a wide range of intermediate hosts. Here, we employ a PCR-based approach to verify the amount and pattern of polymorphism in *eg95*-related genes from 7 *Echinococcus* species. The results are discussed in terms of the potential effect of polymorphism on vaccine efficacy and parasite adaptation.

## Materials and Methods

### Parasite materials

Genomic DNA from *Echinococcus* spp. metacestodes collected from different hosts and geographic regions has been purified and used to build a gDNA library, in collaboration with several research groups, since 10 years ago. The present study includes 27 isolates of *E. granulosus* (haplotype G1 and its variants), 15 of *E. multilocularis*, 7 of *E. canadensis* (haplotypes G6 and G7), 5 of *E. ortleppi* (haplotype G5), 1 *E. equinus*, 1 *E. oligarthrus* and 1 *E. vogeli* from our library. Detailed information about geographic locations, hosts and genotypes are given in Table S1.

### PCR and sequencing

We used two sets of primers in our PCR experiments. One pair (EG95-F GTGTAGAGCATATCTAGCTT and EG95-R TTGCATTGACTTACATGAGA) was designed to amplify the complete CDS of genes *eg95-1*, *eg95-2* and *eg95-3* of *E. granulosus*. These primers also amplified homologous genes of the remaining species, except *E. multilocularis*. To amplify the complete *em95* CDS we used the following primer set: EM95-F GTGTAGAGCATATCTAGTTTG and EM95-R TGCGTCGACTCACATGAGG. Amplification reactions for both sets of primers were made in a volume of 50 µl containing 1.25 U of *Ex Taq* Polymerase (Takara), 1× reaction buffer, 2 mM MgCl_2_, 20 pmol each primer (IDT), 200 µM dNTP and 100–500 ng of template DNA. Primer annealing followed a touchdown strategy, starting with 55°C and decreasing 1°C every two cycles during the first 20 cycles, followed by 15 more cycles at 45°C, always for 30 sec. The first denaturing step (95°C) lasted 5 min, and 30 sec in the remaining cycles. Extension was performed at 72°C for 60 sec in the first 34 cycles and for 5 min in the last cycle. Amplicons were purified and sequenced directly. Stopping the PCR at 35 cycles and sequencing the products directly hinders the influence of artifacts such as heteroduplexes and chimeras in the data, because they occur at a higher rate during the last few cycles [18]. Sequencing was performed by cycle sequencing and migrated in an ABI 3730XL machine (Applied Biosystems). Two additional primers were used in the cycle sequencing reactions, to cover the entire CDS of *eg95* and *em95* with at least two reads: F2 ATATAGTGATGTCCCGTTAC and R2 TAAAATATTCCAGGCCTTT. Sequences were assessed for quality and assembled using the SeqMan tool from the Lasergene software (ver 7.0). All *eg95* sequences showed double-peaks in regions of high quality reads, which were interpreted as single nucleotide polymorphisms (SNPs), and not heterozygosis, because our primers amplify separate *eg95* loci. Therefore, we generated contigs in which the SNPs were represented by IUPAC ambiguity codes.

### Cloning and sequencing

The SNPs could not be phased statistically, because we anticipate that they correspond to variants at separate loci (the sequences of genes *eg95-1*, *2* and *3* differ at only 12 nucleotide positions within the covered region). For this, we cloned the *eg95* PCR products of 2 isolates of *E. granulosus*, 2 of *E. ortleppi* and 2 of *E. canadensis* (see Table S1). These species belong to a phylogenetic cluster usually designated *E. granulosus sensu lato*. Amplification was performed with primers EG95-F and EG95-R using *Pfu* DNA polymerase (Promega) following the manufacturer's recommendations. A reduced number of cycles were used in the PCR (30), to avoid incorporation artifacts. Amplicons were cloned using the Zero Blunt TOPO cloning kit (Invitrogen), and the purified plasmids (Qiagen) from 6–8 isolated colonies per cloning experiment were sequenced with universal primers T3 and T7, and with F2 and R2, as previously described.

### Phylogenetic analyses

Three kinds of phylogenetic analyses were performed to investigate the evolution of the *eg95* multigene family. First, we simply aligned the 59 contigs from our direct sequencing dataset using Clustal X [19], and performed a Neighbor Joining analysis with 1000 bootstrap replicates and the Kimura 2-parameters distance using Mega 4.0 [20]. This analysis aimed to evaluate the amount of *eg95* and *em95* diversity within our sample. Second, we downloaded from the GenBank core sequence database 14 *E. granulosus eg95*-related sequences, 1 *E. multilocularis em95*, and 2 *Taenia ovis* 45W sequences, and aligned them to 9 contigs representative of the major clades identified in the previous Neighbor Joining analysis. We wanted not only to understand how the genes characterized in our study relate to the previously reported members of the gene family, but also to describe their evolutionary pattern. For this, we performed a Bayesian analysis with this alignment using Mr Bayes 3.1 [21]. The analysis ran for 1,000,000 generations, sampling every 100th generation, using the GTR+G+I model. Finally, we identified the unambiguous EG95 and EM95 amino acid changes in the clades leading to each *Echinococcus* species or strain. We aligned the *E. equinus*, *E. multilocularis*, *E. oligarthurs* and *E. vogeli* decuced *eg95* and *em95* mRNA sequences derived from the purified PCR products with Clustal X, and translated them into proteins. Since the direct-PCR sequences from *E. granulosus*, *E. canadensis* and *E. ortleppi* showed ambiguities within the coding region, we added their mRNA sequences encoding distinct protein isoforms, as inferred from the cloning experiments, to the former alignment. A maximum parsimony tree was built using Mega 4.0. The amino acid changes observed along the main braches of this tree were tracked, based on parsimony, using MacClade 4.08.

### Searching for positive selection

Positive selection was investigated on the *eg95* sequence variants isolated with the cloning experiments, to circumvent the problem of ambiguous sites (double-peaks). A site or sequence is assumed to be under positive selection when the ratio of nonsynonymous to synonymous substitutions is greater than one. Two approaches were used to detect positive selection in our study. First, the HyPhy package [22] was used for fitting distinct models of codon substitution [23], [24], on a full-likelihood Neighbor Joining tree. This tree was generated with the alignment of the predicted mRNA sequences encoding the secreted protein (exons 2 and 3) derived from our *eg95* clones. Since some models allow the incorporation of heterogeneous ω ratios (the ratio of nonsynonymous to synonymous substitutions per site) among amino acid sites, it is possible then to infer which sites are under selection using a Bayesian method. This approach was used to investigate selection within *E. granulosus sensu lato*. Another approach was running a sliding window of 50 nucleotides along the mRNA sequences and estimating the average ratio of nonsynonymous to synonymous substitutions inside the window, within (pi(a)/pi(s)) and between (K(a)/K(s)) species, using dnaSP version 4.20 [25]. For this analysis we added the *E. multilocularis* sequence dataset, because it did not contain double-peaks. This allows evaluating whether the selection regimes are similar for *E. granulosus sensu lato* and *E. multilocularis*.

## Results

The EG95 primer set amplified a single fragment ranging between 1,263 (*E. oligarthrus*) and 1,316 bp (*E. vogeli*). For *E. multilocularis*, which required another primer set for successful target amplification, a fragment of 1,279 bp was obtained. We found a high degree of nucleotide sequence variation (our dataset is deposited in GenBank with accessions EU595882–EU595964, and the multiple sequence alignment is shown in Figure S1), which can be summarized as follows. Excepting *E. multilocularis*, all isolates show sites with double-peaks within reads of high quality, which we represented by IUPAC ambiguity codes. Ambiguous sites usually appear consistently, in different isolates, and are more frequent in introns, than in exons (see Table 1). Alignments do not show indels within exons, but introns contain many gaps of different sizes. Two large gaps are found alternatively in two groups of sequences, one inside Intron 1 (45 bp in *E. granulosus*, *E. ortleppi* and *E. canadensis*; 62 bp in *E. equinus*) and another inside Intron 2 (34 bp in *E. multilocularis* and *E. oligarthrus*). *E. vogeli eg95* did not show any gap in our alignment, and *E oligarthrus eg95* contains a different large gap inside Intron 1 (16 bp). The *E. vogeli* gene contains a transversion in Exon I, which generates a stop codon, suggesting that it represents a pseudogene. Ninety two percent of all nucleotide substitutions (57/62) are homogeneously distributed among both introns and Exon 2 (Table 1). Overall, there is a clear two-fold excess of transitions over transversions.

**Table 1:** Number of nucleotide substitutions in the 59 contigs obtained from the direct PCR sequencing, in different regions of the *eg95* gene.

Region	Sites*	Invariant	Transitions	Transversions	Ambiguous	Indels
5′ UTR	All	4	0	0	0	no
Exon 1	All	67	1	1	4	no
	1^st^	23	1	0	2	
	2^nd^	22	0	0	1	
	3^rd^	22	0	0	1	
Intron 1	All	542	14	10	64	yes
Exon 2	All	284	14	5	14	no
	1^st^	96	4	1	2	
	2^nd^	95	5	2	7	
	3^rd^	93	5	2	5	
Intron 2	All	179	10	4	30	yes
Exon 3	All	91	3	0	3	no
	1^st^	30	1	0	1	
	2^nd^	32	0	0	1	
	3^rd^	29	2	0	1	
3′ UTR	All	2	0	0	0	no
	Total	1169	42	20	115	

Invariant = sites showing no changes; transitions = purines changed to purines or pyrimidines to pyrimidines; transversions = purines changed to pyrimidines or vice versa; ambiguous = sites showing double-peaks; indels = deleted sites; UTR = untranslated region.

* 1^st^, 2^nd^, and 3^rd^ refer to codon positions.

**Figure 1:**
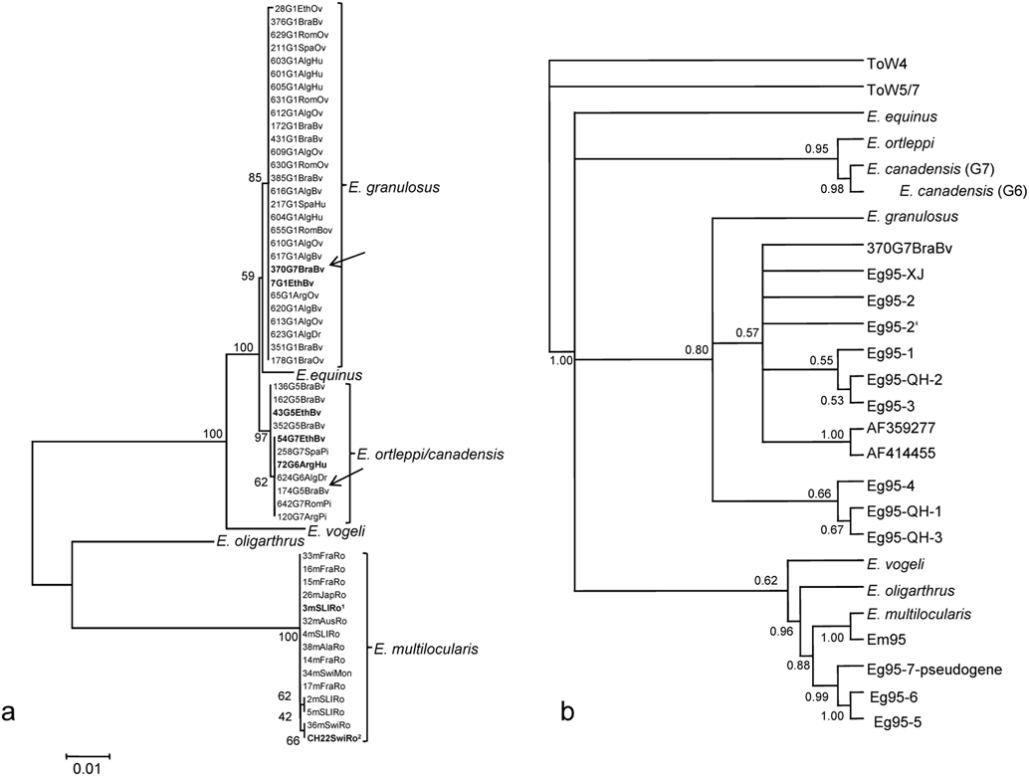
Phylogenetic analyses of *eg95* and *em95* sequences. a) Neighbor Joining tree based on the Kimura 2-parameters distance. Different *E. granulosus*, *E. canadensis* and *E. ortleppi* isolates are identified by a number, followed by the *cox1* haplotype, and abbreviations referring to the geographic origin (Alg = Algeria, Arg = Argentina, Bra = Brazil, Eth = Ethiopia, Rom = Romainia, Spa = Spain) and to the respective host (Bv = cattle, Dr = dromedary, Hu = human, Ov = sheep, Pi = pig); for *E. multilocularis*, only the geographic origin (Aus = Austria, Fra = France; Jap = Japan, SLI = St Laurence Island, Swi = Switzerland) and the host (Mon = monkey, Ro = rodent) are indicated. b) Bayesian phyologeny constructed with model GTR+G+I. A subset of sequences obtained in the present study is compared to GenBank *eg95*-related sequences. ToW4 and ToW5/7 are *Taenia ovis* sequences published by Waterkeyn et al [9]; *eg95-XJ*, *eg95-QH-2*, *eg95-QH-1*, *eg95-QH-3* are unpublished sequences deposited by Lin et al (2002) with accessions AF465599, AY421717, AY421716 and AY421718, respectively; *eg95-2*, *eg95-2′*, *eg95-1*, *eg95-3*, *eg95-4*, *eg95-7*-pseudogene, *eg95-6* and *eg95-5* were obtained by Chow et al [3]; AF359277 and AF414455 were submitted directly to GenBank by Lu et al (2001); and *em95* is the *E. multilocularis* sequence published by Gauci et al [4]. Numbers on each node represent the bootstrap support (a) or the posterior probability (b). Arrows indicate outlier sequences with respect to the mitochondrial *cox1* haplotype (see text).

Our sequences are grouped into three major clusters, corresponding to *E. granulosus*, *E. ortleppi/canadensis* and *E. multilocularis* (Fig. 1a). There is a single case, indicated by an arrow on Fig. 1a, in which an *E. canadensis* isolate showed a heterologous *eg95* sequence pattern, typical of *E. granulosus*. Within each cluster, sequences share 99–100% identity, and *E. equinus*, *E. vogeli* and *E. oligarthrus* sequences fall outside them, although the position of *E. equinus* is not very well supported by bootstrap re-sampling. Within the *E. ortleppi/canadensis* cluster, there seems to be a tendency of differentiation, but this sub-clustering is also not well statistically supported, and contains another outlier (an *E. ortleppi* isolate grouped within the *E. canadensis* sub-cluster, see Fig. 1a).

The Bayesian phylogenetic analysis compared a sample of our sequences to a group of representative GenBank *eg95*-related sequences (Fig. 1b). It was not possible to root the phylogeny using homologous sequences from *Taenia ovis* (ToW4 and ToW5/7), because, although the closest *eg95* relatives, they are too divergent to unambiguously position the root. Additional phylogenetic analyses performed with this dataset using other methods (data not shown), did not help to overcome that difficulty. However, a few conclusions can be drawn from the tree shown in Fig. 1b. *E. oligarthrus* and *E. multilocularis* sequences are more related to the previously described *eg95-5*, *6* and *7* genes, whereas the *E. granulosus* gene (and the *E. canadensis* outlier) groups with *eg95-1*, *2*, *3* and *4*. The positions of *E. vogeli* and *E. equinus* are not very clear, but *eg95* sequences from *E. ortleppi* and *E. canadensis* diverge considerably from the previously described sequences, forming a separate clade with high statistical support.

**Figure 2:**
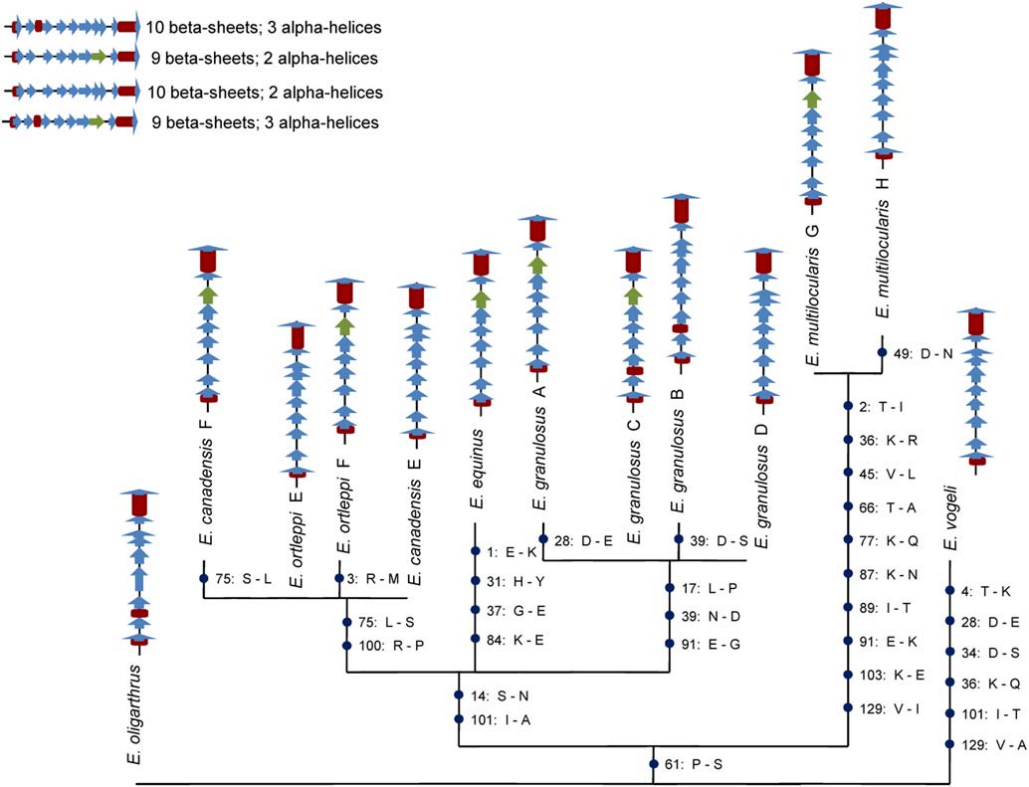
Tracking the unambiguous amino acid replacements along the phylogeny of EG95 isoforms. Particular amino acid changes, and their positions, are indicated on each branch. The predicted secondary structure for each isoform is represented schematically to illustrate evolutionary convergence. Red bars are alpha-helix regions; blue arrows are conserved beta-sheets and green arrows are variant beta sheets.

The variant EG95 amino acid sequences identified in the present study were aligned and used to draw a maximum parsimony tree. One of the 51 equally parsimonious trees is displayed in Fig. 2. The unambiguous amino acid replacements inferred for each branch, and their respective positions, are indicated. We used the PSIPRED method [26] to understand the effect of these replacements in the EG95 secondary structure. The prediction server [27] available at http://bioinf.cs.ucl.ac.uk/psipred/ was used to infer the secondary structure of each isoform. The amino acid replacements observed along the EG95 phylogenetic tree result in a convergent pattern of evolution in terms of protein secondary structure (Fig. 2). For example, the ancestral *E. oligarthrus* isoform containing 10 beta sheets and 3 alpha-helices, is similar to that predicted for isoform *E. granulosus* B, which differs from the former by 7 amino acid replacements. The number of beta-sheets is also convergent in *E. canadensis/ortleppi* F, *E. equinus*, *E. granulosus* A/C and *E. multilocularis* G. It is worth mentioning, additionally, that the distinct isoforms of *E. canadensis*, *E. granulosus* and *E. ortleppi* were obtained from the same isolate, whereas the *E. multilocularis* variants G and H are from different isolates.

**Figure 3:**
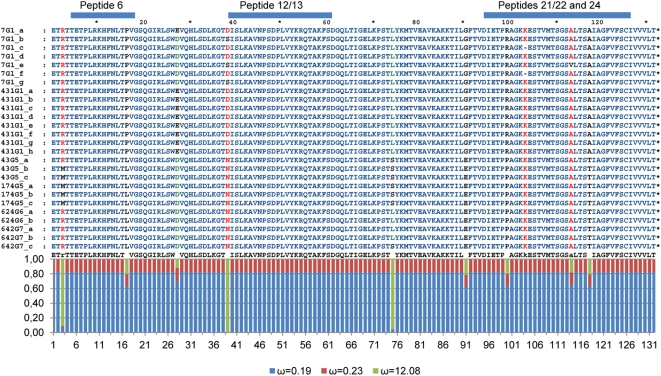
Positive selection analysis of secreted EG95 proteins deduced from nucleotide sequences isolated with the cloning experiments. Proteins derived from distinct mRNAs are identified by the isolate number (see Table S1), *cox1* haplotype, and a letter. The peptides corresponding to vaccine epitopes as described by Woollard et al [12], [15] are shown above the alignment. Below the consensus sequence, a graph shows the posterior probability (y-axis) of each site (x-axis) showing one of three rates of non synonymous to synonymous substitutions (ω), as estimated by model M3 [24].

Two sites were inferred as being positively selected (ω = 12.08) from model M3, with a posterior probability higher than 0.95, using the sequences derived from our cloning experiments (Fig. 3). Sites 39 and 75 are outside the vaccine epitopes described by Woollard et al [12], but seem to influence protein conformation. Replacements at site 39 occur twice in the lineage leading to *E. granulosus*, where a glycosylation site is lost, being replaced by a charged amino acid (N-D), which is again replaced by a hydrophilic amino acid (D-S), in the branch of *E. granulosus* B (Fig. 2). Site 75 also changes twice, but in the *E. canadensis/ortleppi* clade, where a hydrophobic amino acid is first replaced by a hydrophilic amino acid (L-S) and then reverted (S-L) in the branch of *E. canadensis* F. Two other relevant replacements are the following: 1) at site 91 a charged acidic amino acid is replaced by a hydrophobic amino acid in *E. granulosus* (E-G), and by a basic amino acid in *E. multilocularis* (E-K); 2) site100, which is located inside the vaccine epitope, shows a replacement of a charged basic by a hydrophobic amino acid (R-P), in the *E. ortleppi/canadensis* clade (see Fig. 2). Additionally, glycolsylation sites are generated twice along the branch leading to *E. multilocularis* isoforms G and H (sites 49 and 87), and once (site 14) in the clade including *E. granulosus*, *E. equinus*, *E. ortleppi* and *E. canadensis*.

**Figure 4:**
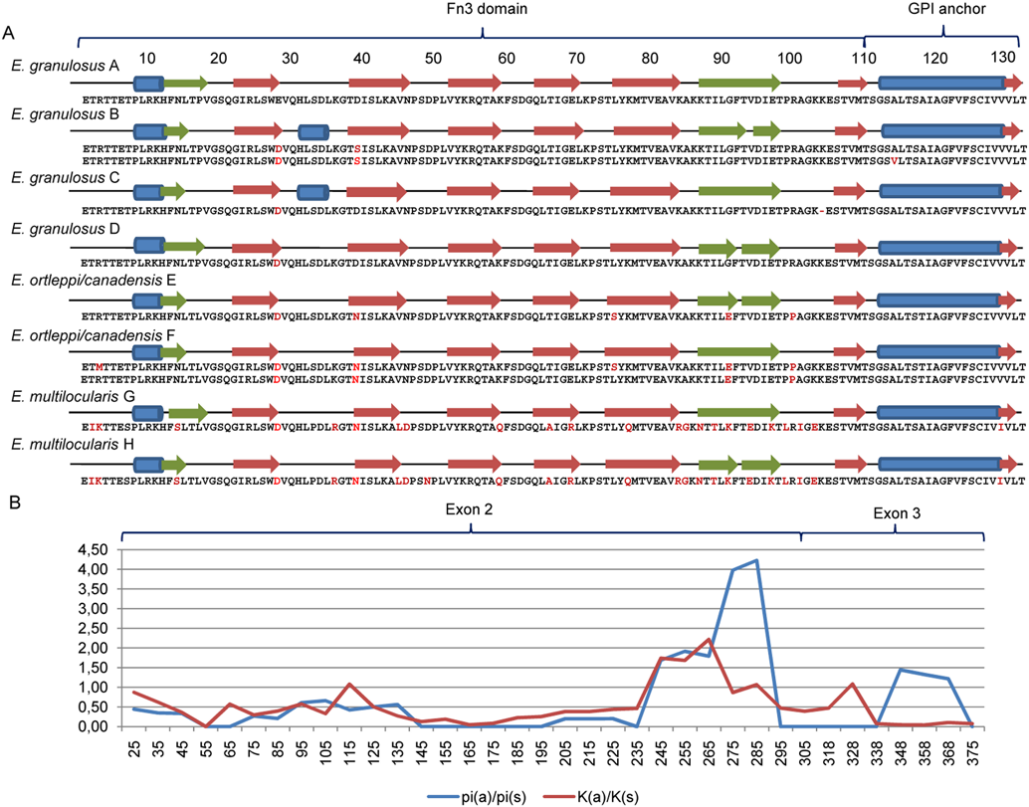
Positive selection analysis of *eg95* and *em95* mRNA sequences encoding the secreted protein. a) Predicted secondary structure of EG95 isoforms. Amino acid differences in relation to *E. granulosus* A are shown in red; blue bars are alpha-helix regions; red arrows are conserved beta-sheets and green arrows are variant beta sheets. The regions corresponding to the fibronectin type III (Fn3) domain and the GPI anchor are indicated. b) Graphic representation of the estimated ratio of non synonymous to synonymous polymorphism within *E. granulosus* sensu lato (pi(a)/pi(s)) and divergence to *E. multilocularis* (K(a)/K(s)) calculated within a sliding window of 50 nucleotides moving in steps of 10 nucleotides along the *eg95* CDS. The nucleotide positions corresponding to exons 1 and 2 are indicated. The downstream region of Exon2, encoding the C-terminal portion of the EG95 and EM95 Fn3 domain, and showing polymorphism in the number of beta-sheets, contains the highest level of non synonymous to synonymous polymorphism and divergence.

Positive selection was further investigated using a sliding window approach, and adding the mRNA sequences of *E. multilocularis* to the alignment containing the distinct *eg95* clones (Fig. 4b). The ratio of nucleotide diversity at nonsynonymous to synonymous sites (pi(a)/pi(s)) is suggestive of positive selection within *E. granulosus sensu lato* (the group including *E. granulosus*, *E. ortleppi* and *E. canadensis*), in the downstream region of Exon II. The pi(a)/pi(s) ratio reaches its maximum ( = 4.23) between nucleotides 261–313 of the mRNA. An overlapping region (sites 241–290) seems under positive selection by calculating the ratio of divergence in nonsynonymous to synonymous sites (K(a)/K(s) = 2.22) between *E. granulosus sensu lato* and *E. multilocularis*. Replacements in these regions seem to alter the number of beta sheets in the C-terminal portion of the EG95 Fn3 domain (Fig. 4a).

## Discussion

### Molecular evolution of the eg95 gene family

Our studies uncovered a large amount of variability in *eg95* genes. The numerous ambiguities in our sequences, and the fact that several genes encoding slightly different isoforms can be isolated from a single metacestode, suggests that the number of *eg95* copies in the *Echinococcus* genome might have been underestimated. Most of the adaptive genetic variation seems to occur within Exon 2 (the number of changes in this region is equivalent to that of introns, see Table 1), which encodes the Fn3 extra-cellular domain of EG95. The large deletions found in introns 1 and 2 of different species and isoforms, may be footprints of sequence rearrangements resulting from unequal crossing over, which is suggestive of gene conversion. Furthermore, the fact that some amino acid sites change repeatedly along the *eg95* phylogeny (28, 39, 75, 91, 101, 129, see Fig. 2) indicates that mutations do not accumulate at random. Indeed, our analyses identified two codons which seem to be under strong positive selection (ω = 12.08): site 39, in the *E. granulosus* lineage, and 75, in the *E. canadensis/ortleppi* lineage. Selection on site 39 is possibly related with the presence of an additional alpha helix at the N-terminal portion of isoforms B and C (see Fig. 4a), and led to the loss of one glycosilation site in EG95 proteins from the entire *E. granulosus* clade. Site 75 could be involved with the evolution of a reduced number of beta sheets in the Fn3 domain C-terminal portion of the *E. canadensis/ortleppi* isoform F. On average, we found that the region encoding the C-terminal portion of the extra-cellular domain (nucleotides 241–313) contains the highest ratio (>1) of nonsyononymous to synonymous polymorphism (pi(a)/pi(s)) and divergence (K(a)/K(s)).

### Positive selection on EG95 and parasite adaptation

*Eg95* variability is found both at the level of the individual (the same metacestode may express slightly distinct isoforms) and of the population (isolates may differ in their *eg95* alleles), indicating a dual role in parasite adaptation. On one side, variability at the individual level allows the parasite to differentially express distinct isoforms according to needs. Distinct isoforms, differing in their secondary structure, may also differ in their ability to bind a variety of receptors from a particular host. Population polymorphism, on the other hand, facilitates parasite adaptation to distinct hosts. We ought to find out what are the cellular mechanisms in which EG95 plays a role, and its receptors, to fully understand the effect of the amino acid changes identified in our study.

Although a considerable amount of genetic variation is found even within single metacestode, *eg95* sequences from each *Echinococcus* spp. are conserved. Indeed, as already described by Chow et al [28], the sequences found for *E. canadensis* isolates showing haplotype G6 (camel strain) are identical to those of haplotype G7 (pig strain). We designed primers to amplify a particular sub-set of family members (*eg95-1*, *2* and *3*), but surprisingly *E. oligarthrus* and *E. vogeli* sequences are more related to a distant clade including *eg95-5*, *6* and *7* (Fig. 1b). This indicates that some of the *eg95* duplication events leading to the distinct sub-families may be very recent. Our findings also indicate recent events of genetic exchange between species, or an ancient polymorphism: one isolate containing a G7 haplotype (identified accordingly as *E. canadensis*) shows an *eg95* sequence similar to *E. granulosus*. Selection seems to drive EG95 evolution by its secondary structure, since the pattern of sequence change is convergent with regard to protein folding (Fig. 2). Moreover, an association exists between the degree of intermediate host specificity and the number of *eg95* variants identified for each species. Species with high host specificity, such as *E. ortleppi* (specifically infects cattle and eventually humans), show a reduced number of double-peaks in the direct PCR sequencing, and a smaller number of isoforms were isolated with the cloning experiment, when compared to *E. granulosus*, which is able to infect a large number of intermediate host species. If EG95 indeed has a role in parasite invasion, having a variety of protein isoforms must be adaptive in terms of the ability to invade different hosts.

### Positive selection on EG95 and vaccine efficacy

The positively selected sites identified in the present study occur outside the vaccine epitopes characterized by Woollard et al [12] (Fig. 3). However, considering that they are conformational [15], we predict that the amino acid changes observed may compromise the efficacy of the vaccine in other species than *E. granulosus*. Nevertheless, it is seen that the evolution of EG95 conformation is convergent. Five of the 7 species analyzed for protein secondary structure in our study (*E. granulosus*, *E. equinus*, *E. ortleppi*, *E. canadensis* and *E. multilocularis*) show an isoform containing 9 beta-sheets and 2 alpha-helices (Fig. 2). This structure, corresponding to isoform *E. granulosus* A, is encoded by the previously characterized gene *eg95-5* (data not shown). However, the vaccine is based on an antigen derived from gene *eg95-1*, which encodes the isoform *E. granulosus* D, with 10 beta sheets. This structure also appears in *E. vogeli*, *E. multilocularis*, *E. granulosus*, *E. ortleppi* and *E. canadensis*. Maximum vaccine efficacy could possibly be obtained by a mixture of both isoforms.

It has to be kept in mind, however, that the evolutionary pattern of the *eg95* gene family strongly suggests a great flexibility. The protein has changed several times along the *Echinococcus* phylogeny, and the additional variant *eg95* copies within a single genome may be differentially expressed. Although no evidences of alternative splicing have been found for *eg95* transcripts, related genes encoding *Taenia solium* 45W antigen were demonstrated to be alternatively spliced [29]. All these mechanisms could be used by the parasite to escape from the vaccine-elicited immune response, and should be the focus of further studies.

## Supporting Information

Table S1 List of *Echinococcus* spp. isolates included in our work. The isolate number corresponds to its assession in our database. The respective genotypes for mitochondrial cytochrome oxydase 1 (*cox1*) and cytosolic malate dehydrogenase (*mdh*) genes are indicated. Excepting *E. multilocularis*, all other species showed double-peaks in regions of high quality reads of eg95, which we interpreted as polymorphisms (SNPs). Therefore, a few isolates were chosen to perform cloning experiments (see Materials and Methods) aiming to characterize their distinct EG95 isoforms.
(0.11 MB DOC)

Figure S1 Alignment of *eg95* sequences analyzed in our study. Sites 1–4 correspond to the 5′UTR, 5–74 to exon 1, 75–698 to intron 1, 699–1004 to exon 2, 1005–1221 to intron 2, 1222–1316 to exon3 and 1317–1318 to 3′UTR.
(4.18 MB RTF)
