# Supplementary material for: The EG95 Antigen of Echinococcus spp. Contains Positively Selected Amino Acids, which May Influence Host Specificity and Vaccine Efficacy
Source: PLoS One. 2009 Apr 29;4(4):e5362. doi: 10.1371/journal.pone.0005362 (PMC2671473; doi:10.1371/journal.pone.0005362)
Supplement: Table S1 — List of Echinococcus spp. isolates included in our work. The isolate number corresponds to its assession in our database. The respective genotypes for mitochondrial cytochrome oxydase 1 (cox1) and cytosolic malate dehydrogenase (mdh) genes are indicated. Excepting E. multilocularis, all other species showed double-peaks in regions of high quality reads of eg95, which we interpreted as polymorphisms (SNPs). Therefore, a few isolates were chosen to perform cloning experiments (see Materials and Methods) aiming to characterize their distinct EG95 isoforms. (0.11 MB DOC) [file pone.0005362.s001.doc]

**Table S1:** List of *Echinococcus* spp. isolates included in our work. The isolate number corresponds to its assession in our database. The respective genotypes for mitochondrial cytochrome oxydase 1 (*cox1*) and cytosolic malate dehydrogenase (*mdh*) genes are indicated. Excepting *E. multilocularis,* all other species showed double-peaks in regions of high quality reads of *eg95*, which we interpreted as polymorphisms (SNPs). Therefore, a few isolates were chosen to perform cloning experiments (see Materials and Methods) aiming to characterize their distinct EG95 isoforms.

| ISOLATE | SPECIES | HOST | LOCATION | *cox1* | *mdh** | EG95 isoforms** |
| --- | --- | --- | --- | --- | --- | --- |
| 601 | *E. granulosus* | Human | Algeria | G1 | Md2/Md2 | - |
| 603 | *E. granulosus* | Human | Algeria | G1 | Md2/Md2 | - |
| 604 | *E. granulosus* | Human | Algeria | G1 | Md1/Md2 | - |
| 605 | *E. granulosus* | Human | Algeria | G1 | Md2/Md2 | - |
| 609 | *E. granulosus* | Sheep | Algeria | G1 | Md1/Md2 | - |
| 610 | *E. granulosus* | Sheep | Algeria | G1 | Md1/Md2 | - |
| 612 | *E. granulosus* | Sheep | Algeria | G1 | Md2/Md2 | - |
| 613 | *E. granulosus* | Sheep | Algeria | G1 | Md1/Md2 | - |
| 616 | *E. granulosus* | Cattle | Algeria | G1 | Md1/Md1 | - |
| 617 | *E. granulosus* | Cattle | Algeria | G1 | Md1/Md2 | - |
| 620 | *E. granulosus* | Cattle | Algeria | G1 | Md2/Md2 | - |
| 623 | *E. granulosus* | Dromedary | Algeria | G1 | Md1/Md2 | - |
| 65 | *E. granulosus* | Sheep | Argentina | G1 | Md2/Md2 | - |
| 172 | *E. granulosus* | Cattle | Brazil | G1 | Md2/Md2 | - |
| 178 | *E. granulosus* | Sheep | Brazil | G1 | - | - |
| 351 | *E. granulosus* | Cattle | Brazil | G1 | Md1/Md2 | - |
| 376 | *E. granulosus* | Cattle | Brazil | G1 | Md2/Md2 | - |
| 385 | *E. granulosus* | Cattle | Brazil | G1 | Md1/Md1 | - |
| 431^§^ | *E. granulosus* | Cattle | Brazil | G1 | Md1/Md1 | A |
| 7^§^ | *E. granulosus* | Cattle | Ethiopia | G1 | - | A, B, C, D |
| 28 | *E. granulosus* | Sheep | Ethiopia | G1 | - | - |
| 629 | *E. granulosus* | Sheep | Romania | G1 | Md2/Md2 | - |
| 630 | *E. granulosus* | Sheep | Romania | G1 | Md2/Md2 | - |
| 631 | *E. granulosus* | Sheep | Romania | G1 | Md2/Md2 | - |
| 655 | *E. granulosus* | Cattle | Romania | G1 | - | - |
| 211 | *E. granulosus* | Sheep | Spain | G1 | - | - |
| 217 | *E. granulosus* | Human | Spain | G1 | - | - |
| 136 | *E. ortleppi* | Cattle | Brazil | G5 | Md3/Md3 | - |
| 162 | *E. ortleppi* | Cattle | Brazil | G5 | - | - |
| 174^§^ | *E. ortleppi* | Cattle | Brazil | G5 | Md3/Md3 | F |
| 352 | *E. ortleppi* | Cattle | Brazil | G5 | Md3/Md3 | - |
| 43^§^ | *E. ortleppi* | Cattle | Ethiopia | G5 | - | E, F |
| 624^§^ | *E. canadensis* | Dromedary | Algeria | G6 | Md3/Md3 | F |
| 72 | *E. canadensis* | Human | Argentina | G6 | Md3/Md3 | - |
| 370 | *E. canadensis* | Cattle | Brazil | G7 | Md3/Md3 | - |
| 54 | *E. canadensis* | Cattle | Ethiopia | G7 | - | - |
| 120 | *E. canadensis* | Pig | Argentina | G7 | - | - |
| 258 | *E. canadensis* | Pig | Spain | G7 | - | - |
| 642^§^ | *E. canadensis* | Pig | Romania | G7 | Md3/Md3 | E, F |
| 252 | *E. equinus* | Horse | Spain | G4 | - | - |
| 2C | *E. oligarthrus* | Rodent | Brazil | O2 | - | - |
| 8H | *E. vogeli* | Human | Brazil | V2 | - | - |
| 2m | *E. multilocularis* | Rodent | St. Laurence Isl. | - | - | G |
| 5m | *E. multilocularis* | Rodent | St. Laurence Isl. | - | - | G |
| 36m | *E. multilocularis* | Rodent | Switzerland | - | - | H |
| CH22 | *E. multilocularis* | Rodent | Switzerland | M3 | - | H |
| 3m | *E. multilocularis* | Rodent | St. Laurence Isl. | - | - | G |
| 4m | *E. multilocularis* | Rodent | St. Laurence Isl. | - | - | G |
| 14m | *E. multilocularis* | Rodent | France | - | - | G |
| 15m | *E. multilocularis* | Rodent | France | - | - | G |
| 16m | *E. multilocularis* | Rodent | France | - | - | G |
| 17m | *E. multilocularis* | Rodent | France | - | - | G |
| 26m | *E. multilocularis* | Rodent | Japan | - | - | G |
| 32m | *E. multilocularis* | Rodent | Austria | - | - | G |
| 33m | *E. multilocularis* | Rodent | France | - | - | G |
| 34m | *E. multilocularis* | Monkey | Switzerland | - | - | G |
| 38m | *E. multilocularis* | Rodent | Alaska | - | - | G |

* According to [1].

** Ascertained for nucleotide sequences showing no double-peaks in the chromatograms.

^§^ Isolates used in cloning experiments.

**Reference:**

[1] [Badaraco JL, Ayala FJ, Bart JM, Gottstein B, Haag KL.](http://www.ncbi.nlm.nih.gov/pubmed/18367173?ordinalpos=2&itool=EntrezSystem2.PEntrez.Pubmed.Pubmed_ResultsPanel.Pubmed_RVDocSum) (2008) Using mitochondrial and nuclear markers to evaluate the degree of genetic cohesion among *Echinococcus* populations. Exp Parasitol 119: 453-459.
