# Supplementary material for: The EG95 Antigen of Echinococcus spp. Contains Positively Selected Amino Acids, which May Influence Host Specificity and Vaccine Efficacy
Source: PLoS One. 2009 Apr 29;4(4):e5362. doi: 10.1371/journal.pone.0005362 (PMC2671473; doi:10.1371/journal.pone.0005362)
Supplement: Figure S1 — Alignment of eg95 sequences analyzed in our study. Sites 1–4 correspond to the 5′UTR, 5–74 to exon 1, 75–698 to intron 1, 699–1004 to exon 2, 1005–1221 to intron 2, 1222–1316 to exon3 and 1317–1318 to 3′UTR. (4.18 MB RTF) [file pone.0005362.s002.rtf]

Figure S1: Alignment of eg95 sequences analyzed in our study. Sites 1-4 correspond to the 5'UTR, 5-74 to exon 1, 75-698 to intron 1, 699-1004 to exon 2, 1005-1221 to intron 2, 1222-1316 to exon3 and 1317-1318 to 3'UTR.

                         10        20        30        40        50        60        70        80        90       100  
                ....|....|....|....|....|....|....|....|....|....|....|....|....|....|....|....|....|....|....|....|
630G1RomOv .    GAAGATGGCATTCCAGTTATGTCTCATTTTGTTTGCGACTTCAGTTTTGGCTCAGGAATACAAAGGAATGGGCGGTAAGTTGCCTTTTAATATTT-GACT 
631G1RomOv .    ..................................................................................YY...........-.... 
623G1AlgDr .    ...............................................................................................-.... 
604G1AlgHu .    ...........................................................................Y..................Y-.... 
620G1AlgBv .    ..............................................................................................Y-.... 
601G1AlgHu .    ..............................................................................................Y-.... 
613G1AlgOv .    ..............................................................................................Y-.... 
7G1EthBv .      .........................................................................R....................Y-.... 
28G1EthOv .     .........................................................................A....................C-.... 
65G1ArgOv .     ..............................................................................................Y-.... 
211G1SpaOv .    ..............................................................................................Y-.... 
217G1SpaHu .    ...............................................................................................-.... 
385G1BraBv .    ..............................................................................................Y-.... 
616G1AlgBv .    ...............................................................................................-.... 
172G1BraBv .    ...............................................................................................-.... 
178G1BraOv .    ...............................................................................................-.... 
612G1AlgOv .    ...............................................................................................-.... 
617G1AlgBv .    ...............................................................................................-.... 
609G1AlgOv .    .........................................................................R..W.................Y-.... 
610G1AlgOv .    .........................................................................R..W.................Y-.... 
431G1BraBv .    .........................................................................R..W.................Y-.... 
351G1BraBv .    ..............................................................................................Y-.... 
603G1AlgHu .    ..............................................................................................Y-.... 
376G1BraBv .    ..............................................................................................Y-.... 
370G7BraBv .    ..............................................................................................Y-.... 
605G1AlgHu .    .........................................................................R....................Y-.... 
629G1RomOv .    ...............................................................................................-.... 
655G1RomBov .   ..............................................................................................Y-.... 
136G5BraBv .    .........................................................................A.....................-.... 
352G5BraBv .    .........................................................................A.....................-.... 
43G5EthBv .     .........................................................................A.....................-.... 
162G5BraBv .    .........................................................................A.....................-.... 
174G5BraBv .    .........................................................................A.....................T.... 
54G7EthBv .     ..........................Y.........................Y....................A.....................-.... 
642G7RomPi .    .........................................................................A.....................-.... 
72G6ArgHu .     .........................................................................A.....................-.... 
120G7ArgPi .    .........................................................................A.....................-.... 
258G7SpaPi .    .........................................................................A.....................-.... 
624G6AlgDr .    .........................................................................R.....................-.... 
Eequinus .      ...............R.........................................................A.....................-.... 
Evogeli .       ---------------------------..............G...............................A.....................-.... 
Eoligarthrus .  ..........C.G..............................A...........A...G.G.....GC....A.....................-.... 
2m .            ............G..............................A..................G.....G....A.....................-.... 
5m .            ............G..............................A..................G.....G....A.....................-.... 
36m .           ............G..............................A..................G.....G....A.....................-.... 
CH22 .          ............G..............................A..................G.....G....A.....................-.... 
3m .            ............G..............................A..................G.....G....A.....................-.... 
4m .            ............G..............................A..................G.....G....A.....................-.... 
14m .           ............G..............................A..................G.....G....A.....................-.... 
15m .           ............G..............................A..................G.....G....A.....................-.... 
16m .           ............G..............................A..................G.....G....A.....................-.... 
17m .           ............G..............................A..................G.....G....A.....................-.... 
26m .           ............G..............................A..................G.....G....A.....................-.... 
32m .           ............G..............................A..................G.....G....A.....................-.... 
33m .           ............G..............................A..................G.....G....A.....................-.... 
34m .           ............G..............................A..................G.....G....A.....................-.... 
38m .           ............G..............................A..................G.....G....A.....................-.... 


                        110       120       130       140       150       160       170       180       190       200         
                ....|....|....|....|....|....|....|....|....|....|....|....|....|....|....|....|....|....|....|....|
630G1RomOv .    CTGATGGGCATTCATACAGAGTATTTGACAGATTGTAGCATATGYTGARTAAAARTTTCATGTACAGCTTCGAATTAAGTTAAAAGTAGTTCCATTCTAC 
631G1RomOv .    .................................................................................................... 
623G1AlgDr .    .................................................................................................... 
604G1AlgHu .    .............................................................................................R...... 
620G1AlgBv .    .................................................................................................... 
601G1AlgHu .    .................................................................................................... 
613G1AlgOv .    .................................................................................................... 
7G1EthBv .      .............................................................................................R...... 
28G1EthOv .     .............................................................................................R...... 
65G1ArgOv .     .............................................................................................R...... 
211G1SpaOv .    .............................................................................................R...... 
217G1SpaHu .    .............................................................................................R...... 
385G1BraBv .    .............................................................................................R...... 
616G1AlgBv .    .............................................................................................R...... 
172G1BraBv .    .................................................................................................... 
178G1BraOv .    .............................................................................................R...... 
612G1AlgOv .    .............................................................................................R...... 
617G1AlgBv .    .............................................................................................R...... 
609G1AlgOv .    .............................................................................................R...... 
610G1AlgOv .    .............................................................................................R...... 
431G1BraBv .    .................................................................................................... 
351G1BraBv .    .................................................................................................... 
603G1AlgHu .    .................................................................................................... 
376G1BraBv .    .............................................................................................R...... 
370G7BraBv .    .............................................................................................R...... 
605G1AlgHu .    .................................................................................................... 
629G1RomOv .    .................................................................................................... 
655G1RomBov .   .................................................................................................... 
136G5BraBv .    ........Y.............................................-...................C.........T............C.. 
352G5BraBv .    ........Y.............................................-...................C.........T............C.. 
43G5EthBv .     ......................................................-...................C.........T............C.. 
162G5BraBv .    ......................................................-...................C.........T............C.. 
174G5BraBv .    ..........................................................................C.........T............C.. 
54G7EthBv .     ..........................................................................C.........T............C.. 
642G7RomPi .    ..........................................................................C.........T............C.. 
72G6ArgHu .     ..........................................................................C.........T............C.. 
120G7ArgPi .    ..........................................................................C.........T............C.. 
258G7SpaPi .    ..........................................................................C.........T............C.. 
624G6AlgDr .    ..........................................................................C.........W............Y.. 
Eequinus .      ..............................R.....................M.....................Y.....Y...W............... 
Evogeli .       ............................................................................................T....... 
Eoligarthrus .  ..................S.................................C.......Y.Y...........A...........C............. 
2m .            .............T.........A..........................T....................A..........T................. 
5m .            .............T.........A..........................T....................A..........T................. 
36m .           .............T.........A..........................T....................A..........T................. 
CH22 .          .............T.........A..........................T....................A..........T................. 
3m .            .............T.........A..........................T....................A..........T................. 
4m .            .............T.........A..........................T....................A..........T................. 
14m .           .............T.........A..........................T....................A..........T................. 
15m .           .............T.........A..........................T....................A..........T................. 
16m .           .............T.........A..........................T....................A..........T................. 
17m .           .............T.........A..........................T....................A..........T................. 
26m .           .............T.........A..........................T....................A..........T................. 
32m .           .............T.........A..........................T....................A..........T................. 
33m .           .............T.........A..........................T....................A..........T................. 
34m .           .............T.........A..........................T....................A..........T................. 
38m .           .............T.........A..........................T....................A..........T................. 


                        210       220       230       240       250       260       270       280       290       300    
                ....|....|....|....|....|....|....|....|....|....|....|....|....|....|....|....|....|....|....|....|
630G1RomOv .    CGCATTTAKGCAAATTGAAACTCTCATTAACCCCATATCGTAGAGGTGTAAGTATCCACACTATTCACTGCTGTTATTTGCTGAGGAGTGAGGTGTATGC 
631G1RomOv .    .................................................................................................... 
623G1AlgDr .    .................................................................................................... 
604G1AlgHu .    .................................................................................................... 
620G1AlgBv .    .................................................................................................... 
601G1AlgHu .    .................................................................................................... 
613G1AlgOv .    .................................................................................................... 
7G1EthBv .      ...................................................................................................S 
28G1EthOv .     ..............................................................R..................................... 
65G1ArgOv .     ................................................................................S.....R......K.....S 
211G1SpaOv .    ...................................................................................................S 
217G1SpaHu .    .................................................................................................... 
385G1BraBv .    .................................................................................................... 
616G1AlgBv .    ........W........................................................................................... 
172G1BraBv .    .................................................................................................... 
178G1BraOv .    .................................................................................................... 
612G1AlgOv .    .................................................................................................... 
617G1AlgBv .    .................................................................................................... 
609G1AlgOv .    .................................................................................................... 
610G1AlgOv .    .................................................................................................... 
431G1BraBv .    .................................................................................................... 
351G1BraBv .    .................................................................................................... 
603G1AlgHu .    .................................................................................................... 
376G1BraBv .    .................................................................................................... 
370G7BraBv .    .................................................................................................... 
605G1AlgHu .    .................................................................................................... 
629G1RomOv .    .................................................................................................... 
655G1RomBov .   .................................................................................................... 
136G5BraBv .    .................................................................................................... 
352G5BraBv .    .................................................................................................... 
43G5EthBv .     .................................................................................................... 
162G5BraBv .    .................................................................................................... 
174G5BraBv .    .................................................................................................... 
54G7EthBv .     .................................................................................................... 
642G7RomPi .    .................................................................................................... 
72G6ArgHu .     .................................................................................................... 
120G7ArgPi .    .................................................................................................... 
258G7SpaPi .    .................................................................................................... 
624G6AlgDr .    .................................................................................................... 
Eequinus .      .......R...........................................................................................- 
Evogeli .       .............................................A.......................A.............................. 
Eoligarthrus .  ............M.......................................................AA..........................G... 
2m .            ......A.......................................................G......A.................T........G... 
5m .            ......A.......................................................G......A.................T........G... 
36m .           ......A.......................................................G......A.................T........G... 
CH22 .          ......A.......................................................G......A.................T........G... 
3m .            ......A.......................................................G......A.................T........G... 
4m .            ......A.......................................................G......A.................T........G... 
14m .           ......A.......................................................G......A.................T........G... 
15m .           ......A.......................................................G......A.................T........G... 
16m .           ......A.......................................................G......A.................T........G... 
17m .           ......A.......................................................G......A.................T........G... 
26m .           ......A.......................................................G......A.................T........G... 
32m .           ......A.......................................................G......A.................T........G... 
33m .           ......A.......................................................G......A.................T........G... 
34m .           ......A.......................................................G......A.................T........G... 
38m .           ......A.......................................................G......A.................T........G... 


                        310       320       330       340       350       360       370       380       390       400   
                ....|....|....|....|....|....|....|....|....|....|....|....|....|....|....|....|....|....|....|....|
630G1RomOv .    TGCACSC---------------------------------------------CTCTACTGGTTAATTACAGTGCCCCAGACGAAAAAATTCGTCACCACT 
631G1RomOv .    .......---------------------------------------------................................................ 
623G1AlgDr .    .......---------------------------------------------...........R................................-... 
604G1AlgHu .    .......---------------------------------------------...........R.....R...Y......................Y... 
620G1AlgBv .    .......---------------------------------------------...........R.....R...Y.......................... 
601G1AlgHu .    .......---------------------------------------------..S........R.........Y.......................... 
613G1AlgOv .    .......---------------------------------------------..S..................Y................Y.....-... 
7G1EthBv .      .......---------------------------------------------.....................Y.......................... 
28G1EthOv .     .......---------------------------------------------..................................T-............ 
65G1ArgOv .     ...M...---------------------------------------------..S........R.....R...Y.............W............ 
211G1SpaOv .    .......---------------------------------------------..S........R.....R...Y.............W............ 
217G1SpaHu .    .......---------------------------------------------..S........R.....R...Y.............W............ 
385G1BraBv .    .......---------------------------------------------..S........R.....R...Y.......................... 
616G1AlgBv .    .......---------------------------------------------..S........R.....R...Y.............W........Y... 
172G1BraBv .    .......---------------------------------------------..G........R.....R...Y.......................... 
178G1BraOv .    .......---------------------------------------------..B........R.....R...Y.......................... 
612G1AlgOv .    .......---------------------------------------------..S........R.........Y.............W............ 
617G1AlgBv .    .......---------------------------------------------..S........R.....R...Y.......................... 
609G1AlgOv .    .......---------------------------------------------...........R.........Y.............W............ 
610G1AlgOv .    .......---------------------------------------------..S........R.....R...Y.............W........Y... 
431G1BraBv .    .......---------------------------------------------...........R.....R...Y.......................... 
351G1BraBv .    .......---------------------------------------------..S........R.................................... 
603G1AlgHu .    .......---------------------------------------------..S........R.....R...Y.......................... 
376G1BraBv .    .......---------------------------------------------..S........R.....R...Y.......................... 
370G7BraBv .    .......---------------------------------------------..S........R.........Y.......................... 
605G1AlgHu .    .......---------------------------------------------..S........R.....R...Y.......................... 
629G1RomOv .    .......---------------------------------------------..S........G.....R...Y.......................... 
655G1RomBov .   .......---------------------------------------------................................................ 
136G5BraBv .    .......---------------------------------------------...........G.........T.......................... 
352G5BraBv .    .......---------------------------------------------...........G.........T.......................... 
43G5EthBv .     .......---------------------------------------------...........G.........T.......................... 
162G5BraBv .    .......---------------------------------------------...........G.........T.......................... 
174G5BraBv .    .......---------------------------------------------...........G.....G...T.......................... 
54G7EthBv .     .......---------------------------------------------...........G.....G...T.......................... 
642G7RomPi .    .......---------------------------------------------...........G.....G...T.......................... 
72G6ArgHu .     .......---------------------------------------------.Y.........G.....G...T.......................... 
120G7ArgPi .    .......---------------------------------------------...........G.....G...T.......................... 
258G7SpaPi .    .......---------------------------------------------...........G.....G...T.......................... 
624G6AlgDr .    .......---------------------------------------------..S........G.....G...T.......................... 
Eequinus .      -------------------------------------------------------------..G.........T.......................... 
Evogeli .       .A.....ACTTTAACGAAAACAGTTAAACATTGCCTGTGATAAAGAAACTGC...........G.........T......................T... 
Eoligarthrus .  .C.....ACTTTAACGAAAACAGGCAAACACYGCTAGYGATAAAGTAACTGC...........G.........T.A.R...........-.......... 
2m .            .......ACTTTAACGAAAACTGTTAAACGTTGCCTGTGATAAAGTGACTGC...........G.........T........................A. 
5m .            .......ACTTTAACGAAAACTGTTAAACGTTGCCTGTGATAAAGTGACTGC...........G.........T........................A. 
36m .           .......ACTTTAACGAAAACTGTTAAACGTTGCCTGTGATAAAGTGACTGC...........G.........T........................A. 
CH22 .          .......ACTTTAACGAAAACTGTTAAACGTTGCCTGTGATAAAGTGACTGC...........G.........T........................A. 
3m .            .......ACTTTAACGAAAACTGTTAAACGTTGCCTGTGATAAAGTGACTGC...........G.........T........................A. 
4m .            .......ACTTTAACGAAAACTGTTAAACGTTGCCTGTGATAAAGTGACTGC...........G.........T........................A. 
14m .           .......ACTTTAACGAAAACTGTTAAACGTTGCCTGTGATAAAGTGACTGC...........G.........T........................A. 
15m .           .......ACTTTAACGAAAACTGTTAAACGTTGCCTGTGATAAAGTGACTGC...........G.........T........................A. 
16m .           .......ACTTTAACGAAAACTGTTAAACGTTGCCTGTGATAAAGTGACTGC...........G.........T........................A. 
17m .           .......ACTTTAACGAAAACTGTTAAACGTTGCCTGTGATAAAGTGACTGC...........G.........T........................A. 
26m .           .......ACTTTAACGAAAACTGTTAAACGTTGCCTGTGATAAAGTGACTGC...........G.........T........................A. 
32m .           .......ACTTTAACGAAAACTGTTAAACGTTGCCTGTGATAAAGTGACTGC...........G.........T........................A. 
33m .           .......ACTTTAACGAAAACTGTTAAACGTTGCCTGTGATAAAGTGACTGC...........G.........T........................A. 
34m .           .......ACTTTAACGAAAACTGTTAAACGTTGCCTGTGATAAAGTGACTGC...........G.........T........................A. 
38m .           .......ACTTTAACGAAAACTGTTAAACGTTGCCTGTGATAAAGTGACTGC...........G.........T........................A. 


                        410       420       430       440       450       460       470       480       490       500   
                ....|....|....|....|....|....|....|....|....|....|....|....|....|....|....|....|....|....|....|....|
630G1RomOv .    GGGTAAAGTCGTTGTCTCCACATTTCATGTGTAGTTCTCTTTCCTT-ACAATTCCTCCAACATTTTATGATYTCTGAACTTTAAATTATTTCACGCACTT 
631G1RomOv .    ..............................................-..................................................... 
623G1AlgDr .    ..............................................-..................................................... 
604G1AlgHu .    ...Y..........................................-..................................................... 
620G1AlgBv .    ..............................................-..................................................... 
601G1AlgHu .    ..............................................-..................................................... 
613G1AlgOv .    ..............................................-..................................................... 
7G1EthBv .      ..............................................-..................................................... 
28G1EthOv .     ..............................................-............................R........................ 
65G1ArgOv .     ..............................................-.................Y..........R........................ 
211G1SpaOv .    ..............................................-............................R........................ 
217G1SpaHu .    ..............................................-............................R........................ 
385G1BraBv .    ...Y..........................................-..................................................... 
616G1AlgBv .    ...Y..........................................-..................................................... 
172G1BraBv .    ..............................................-..................................................... 
178G1BraOv .    ..............................................-..................................................... 
612G1AlgOv .    ..............................................-..................................................... 
617G1AlgBv .    ..............................................-..................................................... 
609G1AlgOv .    ..............................................-............................R........................ 
610G1AlgOv .    ...Y..........................................-............................R........................ 
431G1BraBv .    ...Y..........................................-............................R........................ 
351G1BraBv .    ..............................................-............................R........................ 
603G1AlgHu .    ..............................................-..................................................... 
376G1BraBv .    ..............................................-............................R........................ 
370G7BraBv .    ..............................................-............................R........................ 
605G1AlgHu .    ..............................................-............................R........................ 
629G1RomOv .    ..............................................-..................................................... 
655G1RomBov .   ..............................................-..................................................... 
136G5BraBv .    ......R.......................................-............G.T..............................M....... 
352G5BraBv .    ..............................................-............G.T..............................M....... 
43G5EthBv .     ......R.......................................-............G.T...................................... 
162G5BraBv .    ..............................................-............G.T...................................... 
174G5BraBv .    ..............................................-............G.T....R................................. 
54G7EthBv .     ..............................................-............G.T....R................................. 
642G7RomPi .    ..............................................-............G.T....R................................. 
72G6ArgHu .     ..............................................-............G.T....R................................. 
120G7ArgPi .    ..............................................-............G.T....R................................. 
258G7SpaPi .    ..............................................-............G.T....R................................. 
624G6AlgDr .    ..............................................-............R.W....R................................. 
Eequinus .      ..............................................-............G.T..........Y.....................A..... 
Evogeli .       ..............A...............................T..............T.............................Y.T...... 
Eoligarthrus .  ..............................................CR...........G.T....G............C.................... 
2m .            .A......C.......CG..............G...........C.T............G.T....-..G.....A..T....C................ 
5m .            .A......C.......CG..............G...........C.T............G.T....-..G.....A..T....C................ 
36m .           .A......C.......CG..............G...........C.T............G.T....-..G.....A..T..................... 
CH22 .          .A......C.......CG..............G...........C.T............G.T....-..G.....A..T..................... 
3m .            .A......C.......CG..............G...........C.T............G.T....-..G.....A..T..................... 
4m .            .A......C.......CG..............G...........C.T............G.T....-..G.....A..T..................... 
14m .           .A......C.......CG..............G...........C.T............G.T....-..G.....A..T..................... 
15m .           .A......C.......CG..............G...........C.T............G.T....-..G.....A..T..................... 
16m .           .A......C.......CG..............G...........C.T............G.T....-..G.....A..T..................... 
17m .           .A......C.......CG..............G...........C.T............G.T....-..G.....A..T..................... 
26m .           .A......C.......CG..............G...........C.T............G.T....-..G.....A..T..................... 
32m .           .A......C.......CG..............G...........C.T............G.T....-..G.....A..T..................... 
33m .           .A......C.......CG..............G...........C.T............G.T....-..G.....A..T..................... 
34m .           .A......C.......CG..............G...........C.T............G.T....-..G.....A..T..................... 
38m .           .A......C.......CG..............G...........C.T............G.T....-..G.....A..T..................... 


                        510       520       530       540       550       560       570       580       590       600         
                ....|....|....|....|....|....|....|....|....|....|....|....|....|....|....|....|....|....|....|....|
630G1RomOv .    GCGTATGAYTTTGATCTGCAAATCAACTTGTAGGATAATGCTCATTTTCAGTCCCGTCAAGMAGCTGAATATAGTGATGTCCCGTTACCCATTACTAGTT 
631G1RomOv .    .................................................................................................... 
623G1AlgDr .    .................................................................................................... 
604G1AlgHu .    .................................................................................................... 
620G1AlgBv .    .................................................................................................... 
601G1AlgHu .    .................................................................................................... 
613G1AlgOv .    .................................................................................................... 
7G1EthBv .      .................................................................................................... 
28G1EthOv .     .................................................................................................... 
65G1ArgOv .     .................................................................................................... 
211G1SpaOv .    .................................................................................................... 
217G1SpaHu .    .................................................................................................... 
385G1BraBv .    .................................................................................................... 
616G1AlgBv .    .................................................................................................... 
172G1BraBv .    .................................................................................................... 
178G1BraOv .    .................................................................................................... 
612G1AlgOv .    .................................................................................................... 
617G1AlgBv .    .................................................................................................... 
609G1AlgOv .    .................................................................................................... 
610G1AlgOv .    .................................................................................................... 
431G1BraBv .    .................................................................................................... 
351G1BraBv .    .................................................................................................... 
603G1AlgHu .    .................................................................................................... 
376G1BraBv .    .................................................................................................... 
370G7BraBv .    .................................................................................................... 
605G1AlgHu .    .................................................................................................... 
629G1RomOv .    .................................................................................................... 
655G1RomBov .   .................................................................................................... 
136G5BraBv .    .................................................................................................... 
352G5BraBv .    .................................R.................................................................. 
43G5EthBv .     .................................R.................................................................. 
162G5BraBv .    .................................................................................................... 
174G5BraBv .    .................................................................................................... 
54G7EthBv .     .................................................................................................... 
642G7RomPi .    .................................................................................................... 
72G6ArgHu .     .................................................................................................... 
120G7ArgPi .    .................................................................................................... 
258G7SpaPi .    .................................................................................................... 
624G6AlgDr .    .................................................................................................... 
Eequinus .      AT......................C........................................................................... 
Evogeli .       A................................-.................................................................. 
Eoligarthrus .  A.......................G...................AC................G.A........................-...C...... 
2m .            A..G..C..............................T..............A..A......G.A.A......................-...C.C.... 
5m .            A..G..C..............................T..............A..A......G.A.A......................-...C.C.... 
36m .           A..G..C..............................T..............A..A......G.A.A......................-...C.C.... 
CH22 .          A..G..C..............................T..............A..A......G.A.A......................-...C.C.... 
3m .            A..G..C..............................T..............A..A......G.A.A......................-...C.C.... 
4m .            A..G..C..............................T..............A..A......G.A.A......................-...C.C.... 
14m .           A..G..C..............................T..............A..A......G.A.A......................-...C.C.... 
15m .           A..G..C..............................T..............A..A......G.A.A......................-...C.C.... 
16m .           A..G..C..............................T..............A..A......G.A.A......................-...C.C.... 
17m .           A..G..C..............................T..............A..A......G.A.A......................-...C.C.... 
26m .           A..G..C..............................T..............A..A......G.A.A......................-...C.C.... 
32m .           A..G..C..............................T..............A..A......G.A.A......................-...C.C.... 
33m .           A..G..C..............................T..............A..A......G.A.A......................-...C.C.... 
34m .           A..G..C..............................T..............A..A......G.A.A......................-...C.C.... 
38m .           A..G..C..............................T..............A..A......G.A.A......................-...C.C.... 


                        610       620       630       640       650       660       670       680       690       700         
                ....|....|....|....|....|....|....|....|....|....|....|....|....|....|....|....|....|....|....|....|
630G1RomOv .    CGACCAACATGTTTAGTGCAAATGTGCGCCCTTATCTGCGGTT-TGTGAAACGAAAGGCCTGGAATATTTTAGCTTAATAACATATTTCCAATTTCAGTA 
631G1RomOv .    ...........................................-........................................................ 
623G1AlgDr .    ...........................................-........................................................ 
604G1AlgHu .    .................................R.........-........................................................ 
620G1AlgBv .    ...........................................-........................................................ 
601G1AlgHu .    ...........................................-..............................................R......... 
613G1AlgOv .    ...........................................-..............................................R......... 
7G1EthBv .      ...........................................-........................................................ 
28G1EthOv .     ...........................................-........................................................ 
65G1ArgOv .     ...........................................-........................................................ 
211G1SpaOv .    ...........................................-........................................................ 
217G1SpaHu .    ...........................................-........................................................ 
385G1BraBv .    ...........................................-........................................................ 
616G1AlgBv .    ...........................................-........................................................ 
172G1BraBv .    ...........................................-..............................................R......... 
178G1BraOv .    ...........................................-..K..................................................... 
612G1AlgOv .    ...........................................-..............................................R......... 
617G1AlgBv .    ...........................................-........................................................ 
609G1AlgOv .    Y.................Y........................-..Y..................................................... 
610G1AlgOv .    ...........................................-..Y..................................................... 
431G1BraBv .    Y.................Y........................-..Y...........................Y......................... 
351G1BraBv .    Y..........................................-..Y..................................................... 
603G1AlgHu .    Y..........................................-..Y...........................................R......... 
376G1BraBv .    ...........................................-..Y..................................................... 
370G7BraBv .    Y..........................................-..Y..................................................... 
605G1AlgHu .    ...........................................-..............................................R......... 
629G1RomOv .    ...........................................-........................................................ 
655G1RomBov .   ...........................................-..Y..................................................... 
136G5BraBv .    ..................T.........T.....C........-........................................................ 
352G5BraBv .    ..................T.........T.....C........-........................................................ 
43G5EthBv .     ..................T.........T.....C........-........................................................ 
162G5BraBv .    ..................T.........T.....C........-........................................................ 
174G5BraBv .    ..................T.........T.....C........-.............................T.......................... 
54G7EthBv .     ..................T.........T.....C........-.............................T.......................... 
642G7RomPi .    ..................T.........T.....C........-.............................T.......................... 
72G6ArgHu .     ..................T.........T.....C........-.............................T.......................... 
120G7ArgPi .    ..................T.........T.....C........-.............................T.......................... 
258G7SpaPi .    ..................T.........T.....C........-.............................T.......................... 
624G6AlgDr .    ..................T.........T.....C........-.............................T.......................... 
Eequinus .      ..................T........................-..........M.................R........................... 
Evogeli .       .............C....T........CT....T.......C.A.................A........A............................. 
Eoligarthrus .  ..G...........G...TR.....A.TT....T.B....-----------------...............R..........CG............... 
2m .            ..GT..T.......G...T......A.CT....TCTG.G.C.A-........A...................T..........C...............T 
5m .            ..GT..T.......G...T......A.CT....TCTG.G.C.A-........A...................T..........C...............T 
36m .           ..GT..T.......G...T......A.CT....TCTG.G.C.A-........A...................T..........C...............T 
CH22 .          ..GT..T.......G...T......A.CT....TCTG.G.C.A-........A...................T..........C...............T 
3m .            ..GT..T.......G...T......A.CT....TCTG.G.C.A-........A...................T..........C...............T 
4m .            ..GT..T.......G...T......A.CT....TCTG.G.C.A-........A...................T..........C...............T 
14m .           ..GT..T.......G...T......A.CT....TCTG.G.C.A-........A...................T..........C...............T 
15m .           ..GT..T.......G...T......A.CT....TCTG.G.C.A-........A...................T..........C...............T 
16m .           ..GT..T.......G...T......A.CT....TCTG.G.C.A-........A...................T..........C...............T 
17m .           ..GT..T.......G...T......A.CT....TCTG.G.C.A-........A...................T..........C...............T 
26m .           ..GT..T.......G...T......A.CT....TCTG.G.C.A-........A...................T..........C...............T 
32m .           ..GT..T.......G...T......A.CT....TCTG.G.C.A-........A...................T..........C...............T 
33m .           ..GT..T.......G...T......A.CT....TCTG.G.C.A-........A...................T..........C...............T 
34m .           ..GT..T.......G...T......A.CT....TCTG.G.C.A-........A...................T..........C...............T 
38m .           ..GT..T.......G...T......A.CT....TCTG.G.C.A-........A...................T..........C...............T 


                        710       720       730       740       750       760       770       780       790       800         
                ....|....|....|....|....|....|....|....|....|....|....|....|....|....|....|....|....|....|....|....|
630G1RomOv .    GAGACAAGGACAACAGAGACTCCGCTCCGTAAACACTTCAATTTGACTCCTGTGGGTTCTCAGGGCATTCGCTTAAGTTGGGAAGTCCAACACTTGTCTG 
631G1RomOv .    .................................................................................................... 
623G1AlgDr .    .................................................................................................... 
604G1AlgHu .    .................................................................................................... 
620G1AlgBv .    .................................................................................................... 
601G1AlgHu .    ...................................................................................W..............Y. 
613G1AlgOv .    ...................................................................................W..............Y. 
7G1EthBv .      R..................................................................................W..............Y. 
28G1EthOv .     ...................................................................................T..............Y. 
65G1ArgOv .     ......................................................................R............W..............Y. 
211G1SpaOv .    ......................................................................R............W..............Y. 
217G1SpaHu .    ......................................................................R...........................Y. 
385G1BraBv .    ......................................................................R............W..............Y. 
616G1AlgBv .    ......................................................................R............W..............Y. 
172G1BraBv .    ..................................................................................................Y. 
178G1BraOv .    ...................................................................................W..............Y. 
612G1AlgOv .    ......................................................................R............W..............Y. 
617G1AlgBv .    ......................................................................R............................. 
609G1AlgOv .    ......................................................................R...........................Y. 
610G1AlgOv .    ......................................................................R............W..............Y. 
431G1BraBv .    .................................................................................................... 
351G1BraBv .    ..................................................................................................Y. 
603G1AlgHu .    ..................................................................................................Y. 
376G1BraBv .    .................................................................................................... 
370G7BraBv .    .................................................................................................... 
605G1AlgHu .    .................................................................................................... 
629G1RomOv .    .................................................................................................... 
655G1RomBov .   .................................................................................................... 
136G5BraBv .    .......T.....................C...................T.................................T................ 
352G5BraBv .    .......K.....................C...................T.................................T................ 
43G5EthBv .     .......K.....................C...................T.................................T................ 
162G5BraBv .    .......T.....................C...................T.................................T................ 
174G5BraBv .    .......K.....................C...................T.................................T................ 
54G7EthBv .     .............................C...................T.................................T................ 
642G7RomPi .    .............................C...................T.................................T................ 
72G6ArgHu .     .............................C...................T.................................T................ 
120G7ArgPi .    .............................C...................T.................................T................ 
258G7SpaPi .    .............................C...................T.................................T................ 
624G6AlgDr .    .............................C...................T.................................T................ 
Eequinus .      A................................................T.................................T......T......... 
Evogeli .       ..........A...G.......................T.G........T..........................K...................C..A 
Eoligarthrus .  ....A..A...........G....................G........T.......C.....A.....A.............T................ 
2m .            ....T..A...........G...A................G........T.................................T.....G......C... 
5m .            ....T..A...........G...A................G........T.................................T.....G......C... 
36m .           ....T..A...........G...A................G........T.................................T.....G......C... 
CH22 .          ....T..A...........G...A................G........T.................................T.....G......C... 
3m .            ....T..A...........G...A................G........T.................................T.....G......C... 
4m .            ....T..A...........G...A................G........T.................................T.....G......C... 
14m .           ....T..A...........G...A................G........T.................................T.....G......C... 
15m .           ....T..A...........G...A................G........T.................................T.....G......C... 
16m .           ....T..A...........G...A................G........T.................................T.....G......C... 
17m .           ....T..A...........G...A................G........T.................................T.....G......C... 
26m .           ....T..A...........G...A................G........T.................................T.....G......C... 
32m .           ....T..A...........G...A................G........T.................................T.....G......C... 
33m .           ....T..A...........G...A................G........T.................................T.....G......C... 
34m .           ....T..A...........G...A................G........T.................................T.....G......C... 
38m .           ....T..A...........G...A................G........T.................................T.....G......C... 


                        810       820       830       840       850       860       870       880       890       900         
                ....|....|....|....|....|....|....|....|....|....|....|....|....|....|....|....|....|....|....|....|
630G1RomOv .    ACCTCAAAGGAACAGATATTTCTCTAAAAGCGGTGAATCCTTCTGACCCGTTAGTCTACAAAAGACAAACTGCAAAATTCTCAGATGGACAACTCACTAT 
631G1RomOv .    .................................................................................................... 
623G1AlgDr .    .................................................................................................... 
604G1AlgHu .    .................................................................................................... 
620G1AlgBv .    .................................................................................................... 
601G1AlgHu .    ..............R..................................................................................... 
613G1AlgOv .    ..............RR.................................................................................... 
7G1EthBv .      ..............R..................................................................................... 
28G1EthOv .     .................................................................................................... 
65G1ArgOv .     ..............R..................................................................................... 
211G1SpaOv .    .................................................................................................... 
217G1SpaHu .    .................................................................................................... 
385G1BraBv .    .................................................................................................... 
616G1AlgBv .    ..............RR.................................................................................... 
172G1BraBv .    ..............R..................................................................................... 
178G1BraOv .    ..............R..................................................................................... 
612G1AlgOv .    ..............R..................................................................................... 
617G1AlgBv .    ..............R..................................................................................... 
609G1AlgOv .    .................................................................................................... 
610G1AlgOv .    .................................................................................................... 
431G1BraBv .    .................................................................................................... 
351G1BraBv .    .................................................................................................... 
603G1AlgHu .    ..............R..................................................................................... 
376G1BraBv .    .................................................................................................... 
370G7BraBv .    .................................................................................................... 
605G1AlgHu .    .................................................................................................... 
629G1RomOv .    .................................................................................................... 
655G1RomBov .   .................................................................................................... 
136G5BraBv .    ..............A............................C........................................................ 
352G5BraBv .    ..............A.........................Y..C........................................................ 
43G5EthBv .     ..............A.........................Y..C........................................................ 
162G5BraBv .    ..............A............................C........................................................ 
174G5BraBv .    ..............A............................C........................................................ 
54G7EthBv .     ..............A............................C........................................................ 
642G7RomPi .    ..............A............................C........................................................ 
72G6ArgHu .     ..............A............................C........................................................ 
120G7ArgPi .    ..............A............................C........................................................ 
258G7SpaPi .    ..............A............................C........................................................ 
624G6AlgDr .    ..............A............................C........................................................ 
Eequinus .      .........A....A..................................................................................... 
Evogeli .       G....C........A.......C....................Y....................................C................... 
Eoligarthrus .  ..............A.......C........AA..G.............T...T...............T....C.G...C.G.....R..M.....C.. 
2m .            ......G.......A.......C.........T..G.............CC..........G............C.G.....G............G.C.. 
5m .            ......G.......A.......C.........T..G.............CC..........G............C.G.....G............G.C.. 
36m .           ......G.......A.......C.........T..G........A....CC..........G............C.G.....G............G.C.. 
CH22 .          ......G.......A.......C.........T..G........A....CC..........G............C.G.....G............G.C.. 
3m .            ......G.......A.......C.........T..G.............CC..........G............C.G.....G............G.C.. 
4m .            ......G.......A.......C.........T..G.............CC..........G............C.G.....G............G.C.. 
14m .           ......G.......A.......C.........T..G.............CC..........G............C.G.....G............G.C.. 
15m .           ......G.......A.......C.........T..G.............CC..........G............C.G.....G............G.C.. 
16m .           ......G.......A.......C.........T..G.............CC..........G............C.G.....G............G.C.. 
17m .           ......G.......A.......C.........T..G.............CC..........G............C.G.....G............G.C.. 
26m .           ......G.......A.......C.........T..G.............CC..........G............C.G.....G............G.C.. 
32m .           ......G.......A.......C.........T..G.............CC..........G............C.G.....G............G.C.. 
33m .           ......G.......A.......C.........T..G.............CC..........G............C.G.....G............G.C.. 
34m .           ......G.......A.......C.........T..G.............CC..........G............C.G.....G............G.C.. 
38m .           ......G.......A.......C.........T..G.............CC..........G............C.G.....G............G.C.. 


                        910       920       930       940       950       960       970       980       990       1000        
                ....|....|....|....|....|....|....|....|....|....|....|....|....|....|....|....|....|....|....|....|
630G1RomOv .    CGGCGAACTGAAGCCCTCCACATTATACAAAATGACTGTGGAAGCAGTGAAAGCGAAAAAGACCATTTTGGGATTCACCGTAGACATTGAGACACCGCGC 
631G1RomOv .    .................................................................................................... 
623G1AlgDr .    .................................................................................................... 
604G1AlgHu .    .................................................................................................... 
620G1AlgBv .    .................................................................................................... 
601G1AlgHu .    .................................................................................................... 
613G1AlgOv .    .................................................................................................... 
7G1EthBv .      .................................................................................................... 
28G1EthOv .     .................................................................................................... 
65G1ArgOv .     .................................................................................................... 
211G1SpaOv .    .................................................................................................... 
217G1SpaHu .    .................................................................................................... 
385G1BraBv .    .................................................................................................... 
616G1AlgBv .    .................................................................................................... 
172G1BraBv .    .................................................................................................... 
178G1BraOv .    .................................................................................................... 
612G1AlgOv .    .................................................................................................... 
617G1AlgBv .    .................................................................................................... 
609G1AlgOv .    .................................................................................................... 
610G1AlgOv .    .................................................................................................... 
431G1BraBv .    .................................................................................................... 
351G1BraBv .    .................................................................................................... 
603G1AlgHu .    .................................................................................................... 
376G1BraBv .    .................................................................................................... 
370G7BraBv .    .................................................................................................... 
605G1AlgHu .    .................................................................................................... 
629G1RomOv .    .................................................................................................... 
655G1RomBov .   .................................................................................................... 
136G5BraBv .    ...T...................C...............................................A..........................C. 
352G5BraBv .    ...T...................C...............................................A..........................C. 
43G5EthBv .     ...T...................Y...............................................A..........................C. 
162G5BraBv .    ...T...................C...............................................A..........................C. 
174G5BraBv .    ...T...................Y...............................................A..........................C. 
54G7EthBv .     Y..T...................................................................A..........................C. 
642G7RomPi .    Y..T...................................................................A..........................C. 
72G6ArgHu .     Y..T...................................................................A..........................C. 
120G7ArgPi .    Y..T...................Y...............................................A..........................C. 
258G7SpaPi .    Y..T...................Y...............................................A..........................C. 
624G6AlgDr .    Y..T...................................................................R..........................S. 
Eequinus .      .................................................G.....................A............................ 
Evogeli .       .......................................................................A............T............... 
Eoligarthrus .  ....AG.........T.......................A..........G..G.....GT...C......A........A......AA.A....TC.C. 
2m .            ....AG.........T............C..........A..........G..G......T....C....AA........A......AA.A....TT... 
5m .            ....AG.........T............C..........A..........G..G......T....C....AA........A......AA.A....TT... 
36m .           ....AG.........T............C..........A..........G..G......T....C....AA........A......AA.A....TT... 
CH22 .          ....AG.........T............C..........A..........G..G......T....C....AA........A......AA.A....TT... 
3m .            ....AG.........T............C..........A..........G..G......T....C....AA........A......AA.A....TT... 
4m .            ....AG.........T............C..........A..........G..G......T....C....AA........A......AA.A....TT... 
14m .           ....AG.........T............C..........A..........G..G......T....C....AA........A......AA.A....TT... 
15m .           ....AG.........T............C..........A..........G..G......T....C....AA........A......AA.A....TT... 
16m .           ....AG.........T............C..........A..........G..G......T....C....AA........A......AA.A....TT... 
17m .           ....AG.........T............C..........A..........G..G......T....C....AA........A......AA.A....TT... 
26m .           ....AG.........T............C..........A..........G..G......T....C....AA........A......AA.A....TT... 
32m .           ....AG.........T............C..........A..........G..G......T....C....AA........A......AA.A....TT... 
33m .           ....AG.........T............C..........A..........G..G......T....C....AA........A......AA.A....TT... 
34m .           ....AG.........T............C..........A..........G..G......T....C....AA........A......AA.A....TT... 
38m .           ....AG.........T............C..........A..........G..G......T....C....AA........A......AA.A....TT... 


                        1010      1020      1030      1040      1050      1060      1070      1080      1090      1100        
                ....|....|....|....|....|....|....|....|....|....|....|....|....|....|....|....|....|....|....|....|
630G1RomOv .    GCTGGTAAGTTAGTGT--GTTACCATGGTRTGCAATTCTACGTTAAAGAATATTTCTCACCGACGCCTATCTGCAGTAGTGTTTCCTGTATTTTATTCAA 
631G1RomOv .    ................--.................................................................................. 
623G1AlgDr .    ................--.................................................................................. 
604G1AlgHu .    ................--.................................................................................. 
620G1AlgBv .    ................--.................................................................................. 
601G1AlgHu .    ................--.................................................................................. 
613G1AlgOv .    ................--.................................................................................. 
7G1EthBv .      ................--.................................................................................. 
28G1EthOv .     ................--.................................................................................. 
65G1ArgOv .     ................--.................................................................................. 
211G1SpaOv .    ................--.................................................................................. 
217G1SpaHu .    ................--.................................................................................. 
385G1BraBv .    ................--.................................................................................. 
616G1AlgBv .    ................--.................................................................................. 
172G1BraBv .    ................--.................................................................................. 
178G1BraOv .    ................--.................................................................................. 
612G1AlgOv .    ................--.................................................................................. 
617G1AlgBv .    ................--.................................................................................. 
609G1AlgOv .    ................--.................................................................................. 
610G1AlgOv .    ................--.................................................................................. 
431G1BraBv .    ................--.................................................................................. 
351G1BraBv .    ................--.................................................................................. 
603G1AlgHu .    ................--.................................................................................. 
376G1BraBv .    ................--.................................................................................. 
370G7BraBv .    ................--.................................................................................. 
605G1AlgHu .    ................--.................................................................................. 
629G1RomOv .    ................--.................................................................................. 
655G1RomBov .   ................--.................................................................................. 
136G5BraBv .    .......T........AT.............................................................K.........R.......... 
352G5BraBv .    .......T........AT.............................................................K.........R.......... 
43G5EthBv .     .......T........AT.............................................................K.........R.......... 
162G5BraBv .    .......T........AT.............................................................K.........R.......... 
174G5BraBv .    .......W........AT.............................................................K.........R.......... 
54G7EthBv .     ................AT.................................................................................. 
642G7RomPi .    ................AT.................................................................................. 
72G6ArgHu .     ................AT.................................................................................. 
120G7ArgPi .    ................WT.................................................................................. 
258G7SpaPi .    ................AT...........................................R...............R...................... 
624G6AlgDr .    ................AT.................................................................................. 
Eequinus .      ................AT.................................................Y.........RR..................... 
Evogeli .       A...............AT..........................................................................A....... 
Eoligarthrus .  AT.......C......--..C.T...AC.....---------------------------------T......T.................CA....... 
2m .            AT.......C......--....T...AC.....---------------------------------.......TG.............C..CA....... 
5m .            AT.......C......--....T...AC.....---------------------------------.......TG.............C..CA....... 
36m .           AT.......C......--....T...AC.....---------------------------------.......TG.............C..CA....... 
CH22 .          AT.......C......--....T...AC.....---------------------------------.......TG.............C..CA....... 
3m .            AT.......C......--....T...AC.....---------------------------------.......TG.............C..CA....... 
4m .            AT.......C......--....T...AC.....---------------------------------.......TG.............C..CA....... 
14m .           AT.......C......--....T...AC.....---------------------------------.......TG.............C..CA....... 
15m .           AT.......C......--....T...AC.....---------------------------------.......TG.............C..CA....... 
16m .           AT.......C......--....T...AC.....---------------------------------.......TG.............C..CA....... 
17m .           AT.......C......--....T...AC.....---------------------------------.......TG.............C..CA....... 
26m .           AT.......C......--....T...AC.....---------------------------------.......TG.............C..CA....... 
32m .           AT.......C......--....T...AC.....---------------------------------.......TG.............C..CA....... 
33m .           AT.......C......--....T...AC.....---------------------------------.......TG.............C..CA....... 
34m .           AT.......C......--....T...AC.....---------------------------------.......TG.............C..CA....... 
38m .           AT.......C......--....T...AC.....---------------------------------.......TG.............C..CA....... 


                        1110      1120      1130      1140      1150      1160      1170      1180      1190      1200        
                ....|....|....|....|....|....|....|....|....|....|....|....|....|....|....|....|....|....|....|....|
630G1RomOv .    AGGAAACACTGTTAAACTCACCACCACGAGGCAKTCGTCACTACTCGTTACTATACAGTTTGCATTTGAGTGTCACACATGTYTTGCTTGACWTAATTAG 
631G1RomOv .    .................................................................................................... 
623G1AlgDr .    .................................................................................................... 
604G1AlgHu .    .................................................................................................... 
620G1AlgBv .    .................................................................................................... 
601G1AlgHu .    .................................................................................................... 
613G1AlgOv .    .................................................................................................... 
7G1EthBv .      .....W.............................Y...............................................................R 
28G1EthOv .     .....W.............................Y................................................................ 
65G1ArgOv .     .....W.............................Y................................................................ 
211G1SpaOv .    .....W.............................Y................................................................ 
217G1SpaHu .    .....W.............................Y................................................................ 
385G1BraBv .    .....W.............................Y................................................................ 
616G1AlgBv .    .....W.............................Y................................................................ 
172G1BraBv .    .....W.............................Y................................................................ 
178G1BraOv .    .....W.............................Y................................................................ 
612G1AlgOv .    .....W.............................Y................................................................ 
617G1AlgBv .    .....W.............................Y................................................................ 
609G1AlgOv .    .....W.............................Y................................................................ 
610G1AlgOv .    .....W.............................Y................................................................ 
431G1BraBv .    ...................................Y................................................................ 
351G1BraBv .    .....M.............................Y................................................................ 
603G1AlgHu .    .....W.............................Y................................................................ 
376G1BraBv .    .................................................................................................... 
370G7BraBv .    .................................................................................................... 
605G1AlgHu .    .................................................................................................... 
629G1RomOv .    .................................................................................................... 
655G1RomBov .   .................................................................................................... 
136G5BraBv .    ...........................R........................T.G........GC..........................T........ 
352G5BraBv .    ...........................R........................T.G........GC..........................T........ 
43G5EthBv .     ...........................R........................T.G........GC..........................T........ 
162G5BraBv .    ...........................A........................T.G........GC..........................T........ 
174G5BraBv .    ...........................A........................T.G........GC..........................T........ 
54G7EthBv .     ...........................A........................T.G........GC..........................T........ 
642G7RomPi .    ...........................A........................T.G........GC..........................T........ 
72G6ArgHu .     ...........................A........................T.G........GC..........................T........ 
120G7ArgPi .    ...........................A........................T.G........GC..........................T........ 
258G7SpaPi .    ...........................A........................T.G........GC..........................T........ 
624G6AlgDr .    ...........................R........................W.R........RY..........................Y........ 
Eequinus .      ...........................A........................W.R........RY................................... 
Evogeli .       ..........K...............................................................G......................C.. 
Eoligarthrus .  G.A......C.......CT........T.CA........G.C..C.C....C.....A...A..........A.G.R....................... 
2m .            T.A.......A......CT........T.CA.......TG.C....C....C.........A..........A.G.G.........T............. 
5m .            T.A.......A......CT........T.CA.......TG.C....C....C.........A..........A.G.G.........T............. 
36m .           T.A.......A......CT........T.CA.......TG.C....C....C.........A..........A.G.G.........T............. 
CH22 .          T.A.......A......CT........T.CA.......TG.C....C....C.........A..........A.G.G.........T............. 
3m .            T.A.......A......CT........T.CA.......TG.C....C....C.........A..........A.G.G.........T............. 
4m .            T.A.......A......CT........T.CA.......TG.C....C....C.........A..........A.G.G.........T............. 
14m .           T.A.......A......CT........T.CA.......TG.C....C....C.........A..........A.G.G.........T............. 
15m .           T.A.......A......CT........T.CA.......TG.C....C....C.........A..........A.G.G.........T............. 
16m .           T.A.......A......CT........T.CA.......TG.C....C....C.........A..........A.G.G.........T............. 
17m .           T.A.......A......CT........T.CA.......TG.C....C....C.........A..........A.G.G.........T............. 
26m .           T.A.......A......CT........T.CA.......TG.C....C....C.........A..........A.G.G.........T............. 
32m .           T.A.......A......CT........T.CA.......TG.C....C....C.........A..........A.G.G.........T............. 
33m .           T.A.......A......CT........T.CA.......TG.C....C....C.........A..........A.G.G.........T............. 
34m .           T.A.......A......CT........T.CA.......TG.C....C....C.........A..........A.G.G.........T............. 
38m .           T.A.......A......CT........T.CA.......TG.C....C....C.........A..........A.G.G.........T............. 


                        1210      1220      1230      1240      1250      1260      1270      1280      1290      1300        
                ....|....|....|....|....|....|....|....|....|....|....|....|....|....|....|....|....|....|....|....|
630G1RomOv .    CYGAATGTCTTGTTTACATAGGCAAGAAGGAAAGCACTGTAATGACTAGTGGATCCGCCTTAACATCCGCAATCGCTGGTTTYGTATTCAGCTGCATAGT 
631G1RomOv .    .................................................................................................... 
623G1AlgDr .    .................................................................................................... 
604G1AlgHu .    .................................................................................................... 
620G1AlgBv .    .................................................................................................... 
601G1AlgHu .    .................................................................................................... 
613G1AlgOv .    .................................................................................................... 
7G1EthBv .      .................................................................................................... 
28G1EthOv .     .................................................................................................... 
65G1ArgOv .     .................................................................................................... 
211G1SpaOv .    .................................................................................................... 
217G1SpaHu .    .................................................................................................... 
385G1BraBv .    .......................................................................................-............ 
616G1AlgBv .    .................................................................................................... 
172G1BraBv .    .................................................................................................... 
178G1BraOv .    .................................................................................................... 
612G1AlgOv .    .................................................................................................... 
617G1AlgBv .    .................................................................................................... 
609G1AlgOv .    .................................................................................................... 
610G1AlgOv .    .................................................................................................... 
431G1BraBv .    .................................................................................................... 
351G1BraBv .    .................................................................................................... 
603G1AlgHu .    .................................................................................................... 
376G1BraBv .    .................................................................................Y.................. 
370G7BraBv .    .................................................................................................... 
605G1AlgHu .    .................................................................................................... 
629G1RomOv .    .................................................................................................... 
655G1RomBov .   .................................................................................................... 
136G5BraBv .    ....................................................................A............................... 
352G5BraBv .    ....................................................................A............................... 
43G5EthBv .     ....................................................................A............................... 
162G5BraBv .    ....................................................................A............................... 
174G5BraBv .    ....................................................................A............................... 
54G7EthBv .     ....................................................................A............................... 
642G7RomPi .    ....................................................................A............................... 
72G6ArgHu .     ....................................................................A............................... 
120G7ArgPi .    ....................................................................A............................... 
258G7SpaPi .    ....................................................................A............................... 
624G6AlgDr .    ....................................................................R............................... 
Eequinus .      ........Y...........................................................A............................... 
Evogeli .       ....................................................................A............................... 
Eoligarthrus .  ..CC........................A.C.....TC.............A........................C.....................T. 
2m .            ..CC......C............G....A........C......................................C........G.............. 
5m .            ..CC......C............G....A........C......................................C........G.............. 
36m .           ..CC......C............G....A........C......................................C........G.............. 
CH22 .          ..CC......C............G....A........C......................................C........G.............. 
3m .            ..CC......C............G....A........C......................................C........G.............. 
4m .            ..CC......C............G....A........C......................................C........G.............. 
14m .           ..CC......C............G....A........C......................................C........G.............. 
15m .           ..CC......C............G....A........C......................................C........G.............. 
16m .           ..CC......C............G....A........C......................................C........G.............. 
17m .           ..CC......C............G....A........C......................................C........G.............. 
26m .           ..CC......C............G....A........C......................................C........G.............. 
32m .           ..CC......C............G....A........C......................................C........G.............. 
33m .           ..CC......C............G....A........C......................................C........G.............. 
34m .           ..CC......C............G....A........C......................................C........G.............. 
38m .           ..CC......C............G....A........C......................................C........G.............. 


                        1310     
                ....|....|....|...
630G1RomOv .    GGTTGTCCTTACTTGAAC 
631G1RomOv .    .................. 
623G1AlgDr .    .................. 
604G1AlgHu .    .................. 
620G1AlgBv .    .................. 
601G1AlgHu .    .................. 
613G1AlgOv .    .................. 
7G1EthBv .      .................. 
28G1EthOv .     .................. 
65G1ArgOv .     .................. 
211G1SpaOv .    .................. 
217G1SpaHu .    .................. 
385G1BraBv .    .................. 
616G1AlgBv .    .................. 
172G1BraBv .    .................. 
178G1BraOv .    .................. 
612G1AlgOv .    .................. 
617G1AlgBv .    .................. 
609G1AlgOv .    .................. 
610G1AlgOv .    .................. 
431G1BraBv .    .................. 
351G1BraBv .    .................. 
603G1AlgHu .    .................. 
376G1BraBv .    .................. 
370G7BraBv .    .................. 
605G1AlgHu .    .................. 
629G1RomOv .    .................. 
655G1RomBov .   .................. 
136G5BraBv .    .................. 
352G5BraBv .    .................. 
43G5EthBv .     .................. 
162G5BraBv .    .................. 
174G5BraBv .    .................. 
54G7EthBv .     .................. 
642G7RomPi .    .................. 
72G6ArgHu .     .................. 
120G7ArgPi .    .................. 
258G7SpaPi .    .................. 
624G6AlgDr .    .................. 
Eequinus .      ................G. 
Evogeli .       ..C............... 
Eoligarthrus .  .................. 
2m .            AA................ 
5m .            AA................ 
36m .           AA................ 
CH22 .          AA................ 
3m .            AA................ 
4m .            AA................ 
14m .           AA................ 
15m .           AA................ 
16m .           AA................ 
17m .           AA................ 
26m .           AA................ 
32m .           AA................ 
33m .           AA................ 
34m .           AA................ 
38m .           AA................ 
